# Cystic lymphangioma in adult—a rare case scenario or a misdiagnosis?

**DOI:** 10.1093/jscr/rjab062

**Published:** 2021-03-13

**Authors:** J V Pranav Sharma, Farah Naaz Kazi

**Affiliations:** Surgery, Vydehi Institute of Medical Sciences and Research Centre, Bangalore, India; Surgery, Vydehi Institute of Medical Sciences and Research Centre, Bangalore, India

## Abstract

Cystic lymphangiomas are a rare entity in adults. It is commonly congenital due to obstruction in the lymphatic drainage. We report the case of a 45-year-old female who was admitted with complaints of a lateral neck swelling associated with multiple palpable cervical lymph nodes. Patient was evaluated and relevant investigations were carried out. Her radiological investigations reported conflicting results of lymphatic cyst, branchial cyst and infected cyst or abscess. However, after an excisional biopsy when the sample tissue was sent for histopathology, the results featured a lymphangioma, which is a rare finding in adults of this age.

## INTRODUCTION

Lymphangiomas are deformities of the lymphatic system characterized by lesions that are thin-walled cysts. The lymphatic system is a connection responsible for reallocating extra fluid from tissues and lymph nodes to the venous channels, which detect any microorganism or trigger agents. Just as haemangiomas occur in blood vessels, lymphangiomas occur in lymphatics. It was first described by Redenbacher in 1828 [1]. These malformations can occur at any age, but 90% of these occur in the age group <2 years and usually involves head and neck. It is usually a congregate of dilated lymph sacs occurring in the skin and subcutaneous tissue, which has failed to join the normal lymphatic system during the growth period [2]. They could be microcystic (volume <2 cc) or macrocystic (volume >2 cc). Lymphangiomas can be acquired or congenital. Acquired lymphangiomas can result from trauma, inflammation or lymphatic obstructions, whereas congenital lymphangiomas are more often associated with Turner syndrome.

**Figure 1:**
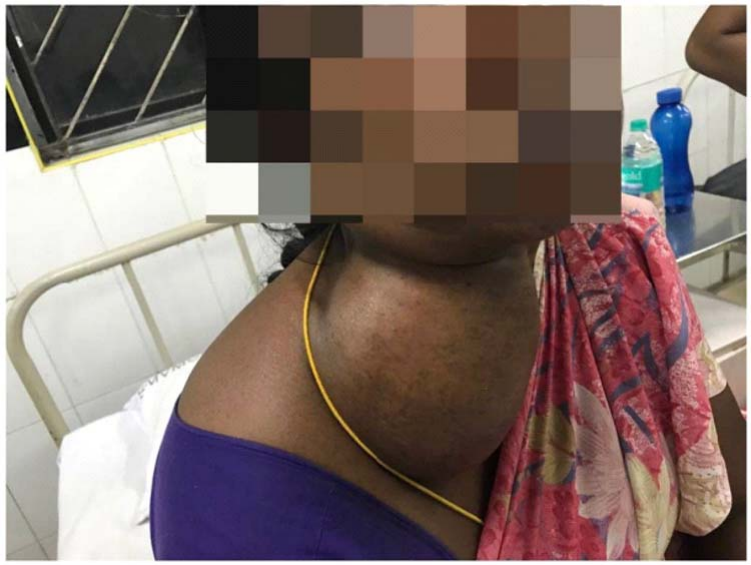
Lateral mass in the neck.

**Figure 2:**
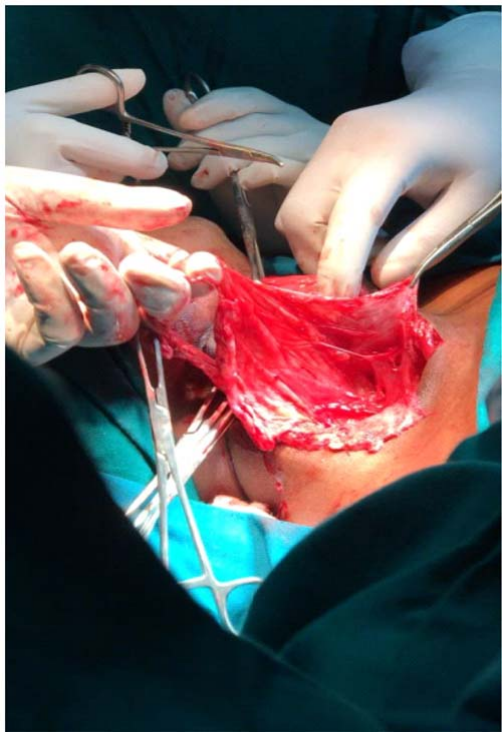
Image showing dissection of the tissues around the mass.

**Figure 3:**
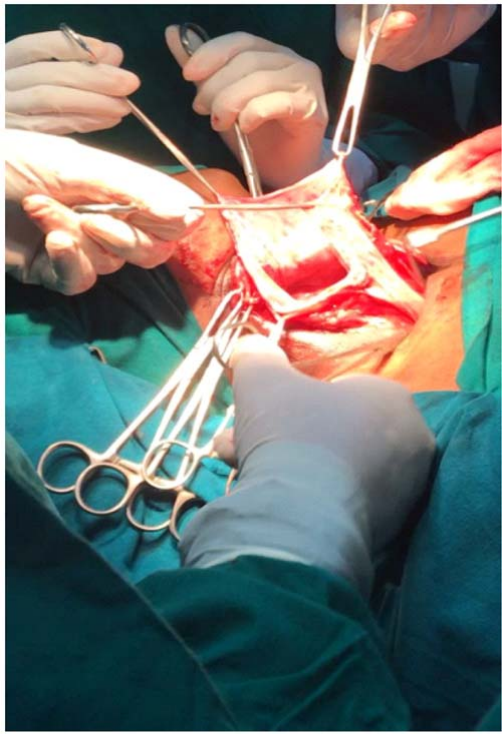
Image showing approach to the mass.

**Figure 4:**
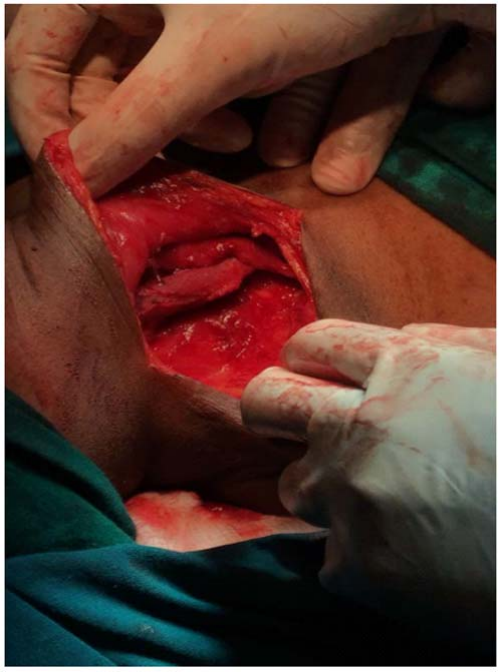
Image showing the mass after dissection.

They could also be capillary or cavernous or cystic. Capillary lymphangiomas are present in the form of skin vesicles at the junction of the body to the extremities like axilla, groin and buttock, whereas cavernous lymphangiomas are classically present as lobulated, transilluminant swellings [3]. Capillary lymphangiomas, as the name suggests, are made up of capillary-sized lymphatic channels, whereas the cavernous lymphangiomas comprise of more dilated lymphatic vessels. Cystic hygroma or lymphangioma, which is generally congenital, can occur in any part of the body, but most commonly occurs in the head and neck region [4]. Cystic lymphangiomas consist of large macrocystic lymphatic channels with protein-rich fluid. These lymphangiomas generally do not have any family history. They generally present as asymptomatic, slow growing masses.

## CASE REPORT

A 45-year-old female presented with complaints of swelling over the right lateral aspect of neck for the last 10 years associated with mild pain over the swelling. Occasionally, the patient also complained of difficulty in breathing and restricted opening of her mouth. She did not complain of any difficulty in swallowing. There was no history of any previous infection like fever, tooth ache or trauma or any other medical condition. Initially, the swelling was small in size and gradually progressed to a size of 10 × 12 cm, extending 4 cm below the lower border of mandible to upper border of clavicle, medial border being 4 cm lateral to midline and lateral border extending up to posterior triangle. On examination, the patient was pale and the swelling was measured to be of size 10 × 12 cm over the right lateral aspect of neck, which did not move on deglutition, was soft and cystic in nature, mobile, non-tender, no local rise in temperature, lateral border of the swelling was extending up to the posterior triangle, and medial border and superior border could not be made out, whereas the inferior border extended up to the clavicle. There was no discharge, redness, sinus from swelling and the overlying skin appeared normal. On intraoral examination, no abnormal findings were noted. Multiple palpable lymph nodes in the cervical region were noted. The nerve supply seemed to be in order.

The baseline investigations for complete blood count, routine urine test and renal function test were reported within normal limits. Ultrasonography of the neck revealed a large cystic swelling of size 13 × 9 × 8 cm (419 cc volume) on the right cervical region extending from the level of hyoid bone to clavicle, with external echoes, no vascularity and no calcification. Carotid vessels were pushed medially in inferior aspect and posteriorly in superior aspect. Multiple enlarged nodes in the cervical region were present with the largest measuring 1.2 cm. The impression was reported as an infected cyst/abscess in right cervical region. A magnetic resonance imaging (MRI) of neck was also performed, which showed a well-defined cystic lesion 13 × 8 × 8 cm extending from level of mandible to right supraclavicular fossa, displacing larynx and trachea to left, carotid vessel displaced medially. The impression was reported as a second branchial cyst or lymphatic cyst.

The plan for this patient was excision biopsy as there was uncertainty in the diagnosis as well as prognosis with a horizontal incision 3 cm below the mandible (Risdon’s incision). This involved complete removal of vesicles in order to prevent any further recurrences, although the risk of complications like scar and disfigurement significantly rises [5]. The excision was done under general anaesthesia and the tissue sample sent for evaluation. The biopsy specimen measured about 14 × 9 × 8 cm with irregular borders and soft in consistency.

Post-operative period was uneventful. There was no neurological impairment. Patient was on antibiotics, analgesics and proton pump inhibitors. Biopsy report confirmed the diagnosis as lymphangioma. It had clusters of lymphoid tissue, large underdeveloped lymphatic channels with unorganized smooth muscle cells in its walls surrounded by connective tissue. It also contained vascular spaces lined by epithelial cells, which were thickened in some places [6].

## DISCUSSION

Lymphangiomas are the lymphatic malformation commonly seen on the neck; however, uncommon in adults and the exact aetiology of which remains unclear in adults. It is often confused with branchial cyst, thymic cyst, pericardial cyst, cystic teratoma and bronchogenic cyst. Generally, all the lymphangiomas remain asymptomatic with no sex predilection. They are soft and progressively growing masses, which may cause compressive symptoms when encroaching surrounding structures of the neck.

Our case was an atypical one as the patient presented with a single large swelling on the side of the neck with no previous history of trauma or infection. The patient underwent an excisional biopsy to reach the final diagnosis.

The patient was followed up for 4 months and patient was doing better. As both the radiological and clinical results were conflicting and amongst the radiological investigations, none came to a single diagnosis, the case was diagnosed clinically as lymphatic cyst with the cystic nature of the swelling and the upper borders, which remained non-palpable. Later the swelling was excised under general anaesthesia and the specimen was sent to histopathology for confirming the diagnosis as cystic lymphangioma. Hence, histopathology proved to be useful in reaching a final diagnosis.

Although clinical evaluations of such swellings may lead to a diagnosis, but it may need further investigations to distinguish it from other types of neck swellings [7]. Radiological investigations like ultrasonography or MRI can be employed; however, sometimes the impression of these reports may delineate one from the diagnosis. Hence, histopathology of such swellings may be a wise investigation, which will lead to the correct diagnosis. Furthermore, the treatment of such swellings depends on the size and depth. Surgical excision is one of the most common plan of care. However, sclerotherapy and laser may also be used [8]. The recurrence rate of such swellings is about 15%; hence, an appropriate method of treatment must be chosen. Due to the size of the swelling in this case, surgical intervention seemed to be the best option; however, some surgeons may prefer a more radical approach in large encroaching swellings [9].

The patient was regularly followed up and looked out for any signs of recurrence, which in any case could be treated by marginal or segmental resection.

In conclusion, lymphangiomas are common in paediatric age group and a rare entity in older age group. Owing to the vague clinic-radiographic characteristics, it is frequently misdiagnosed. However, surgical excision and histopathology study of the specimen is the only option for confirming the diagnosis. In this case, the radiological report ruled out the malignancy and gave diagnosis of lymphatic cyst. However, the histopathology reports revealed the swelling to be lymphangioma. Patient was followed up for 4 months continuously and was stable. Also, this highlights the radiological reports that led to the misdiagnosis. Radiographic reports although more frequently relied on by surgeons proved to be deceiving. The gold standard, hence, for diagnosis of lymphangiomas remains histopathology and cytology study along with long-term follow-up for recurrences.

The case report also highlights the difference in the diagnosis when comparing the histopathological and radiological reports. This makes the diagnosis of cystic lymphangioma in adults all the more challenging.

## CONSENT

A written and informed consent was taken from the patient for the publication of her case as a report.

## CONFLICT OF INTEREST STATEMENT

None declared.
